# Protective Effects of *Spirulina maxima* against Blue Light-Induced Retinal Damages in A2E-Laden ARPE-19 Cells and Balb/c Mice

**DOI:** 10.3390/nu14030401

**Published:** 2022-01-18

**Authors:** Hye-Mi Cho, Ye-Dam Jo, Se-Young Choung

**Affiliations:** 1 Department of Biomedical and Pharmaceutical Sciences, Graduate School, Kyung Hee University, 26, Kyungheedae-ro, Dongdaemun-gu, Seoul 02447, Korea; hyemb2@khu.ac.kr; 2 Department of Life and Nanopharmaceutical Sciences, Graduate School, Kyung Hee University, 26, Kyungheedae-ro, Dongdaemun-gu, Seoul 02447, Korea; whdpeka1004@naver.com; 3 Department of Preventive Pharmacy and Toxicology, College of Pharmacy, Kyung Hee University, 26, Kyungheedae-ro, Dongdaemun-gu, Seoul 02447, Korea

**Keywords:** *Spirulina maxima*, age-related macular degeneration, A2E, blue light, inflammation, oxidative stress

## Abstract

Age-related macular degeneration (AMD) is a significant visual impairment in older people, and there is no treatment for dry AMD. *Spirulina maxima* (*S. maxima*), a cyanobacterium, has inhibitory effects against oxidative stress. However, the protective effects of *S. maxima* and its underlying mechanisms on blue light (BL)-caused macular degeneration are unknown. We aimed to investigate the protective effects of *S. maxima* on blue light-caused retinal damage and demonstrate its underlying mechanisms in human retinal pigment epithelial (ARPE-19) cells and Balb/c retinas. Additionally, the active component of *S. maxima* was examined in the RPE cells. *In vitro*, *S. maxima* decreased BL-induced RPE cell death by inhibiting reactive oxygen species (ROS) production. *S. maxima* inhibited BL-induced inflammation via regulating the NF-κB pathway, inflammatory-related gene expression, and the apoptosis pathway in RPE cells. *In vivo*, administration of *S. maxima* inhibited BL-induced retinal degeneration by restoring the thicknesses of whole retina, ONL (outer nuclear layer), INL (inner nuclear layer), and PL (photoreceptor layer) by BL exposure. Phycocyanin exerted protective effects in the pre-and post-treatment system. Therefore, *S. maxima* could be a potential nutraceutical approach to intercept the patho-physiological processes leading to dry AMD and advancement to wet AMD. Moreover, phycocyanin was a major active compound of *S. maxima*. These findings need to be investigated in human studies, particularly through a clinical trial.

## 1. Introduction

The usage of electronic devices, including television, computers, and smartphones, has risen, resulting in increased exposure to blue light (BL) [1,2]. In healthy eyes, the lens absorbs most light within the ultraviolet spectrum while visible light can reach the retinas and damage eyes. As blue light has relatively shorter wavelengths and higher energy in the visible light spectrum, it is mainly known that BL damages ocular tissues, including retinas and lens, causing several ocular diseases such as macular degeneration, cataracts, and xerophthalmia [3].

Age-related macular degeneration (AMD) is a degenerative eye disease in the elderly characterized by deposited drusen, abnormal function of retinal pigment epithelial (RPE) cells, and abnormal neovascularization causing impaired vision. The previous study reported that AMD caused 22.9% of vision loss among white people in the United States [4]. Another study showed that the AMD prevalence is of 6.6% in South Korea [5]. In dry AMD, yellow drusen are deposited in Bruch’s membrane, and it consists of cellular deposits such as cellular debris of organelles and lipofuscins [6]. In the macula, RPE cells are crucial for assisting photoreceptor cells in performing the normal visual function. In photoreceptor cells, all-trans-retinal is formed by the visual cycle and then reacts with phosphatidylethanolamine to synthesize *N*-retinylidene-*N*-retinylethanolamine (A2E) [2]. RPE cells phagocytose the photoreceptor outer segments. A2E, which a constituent of RPE lipofuscin, accumulated when RPE failed to phagocytosis [7]. Dry AMD can evolve in a progressive form called wet AMD when choroidal neovascularization occurs [8]. The treatment of injecting anti-vascular endothelial growth factor (VEGF) can be performed to delay vision impairments caused by neovascularization [9].

The risk factors for AMD include increased age, obesity, hypertension, and exposure to light. Among them, an increase in age is a crucial risk factor for developing AMD [10]. Lipofuscins, including A2E, are accumulated with age and mediate damage by light exposure because A2E is easily oxidized to oxo-A2E and singlet oxygen when irradiated with blue light [11]. Lipofuscin accumulation causes oxidative stress, which contributes to protein, DNA, and lipid damage and leads to RPE and photoreceptor cell death [12]. Several studies have demonstrated that blue light leads to cell death through generating oxidative forms of A2E and reactive oxygen species (ROS) [13,14]. Our previous study showed that BL irradiation caused increased apoptosis in A2E-loaded RPE cells [15]. We established an animal model for AMD in which intensive BL irradiation was used to induce retinal degeneration in mice [16]. Blue light exposure caused the decreased thickness of the outer nuclear layer (ONL) in mice retina, indicating that apoptosis and the loss of photoreceptor cells occurred [17].

Lutein, one of the carotenoid pigments of the retina, is reported as a preventive substance for retinal degeneration. Moreover, it is widely used clinically around the world [18,19], and has had protective and antioxidant effects in ARPE-19 cells and mouse models [20]. Therefore, we chose lutein as a positive control. However, currently, there is no treatment for dry AMD. Therefore, the development of preventive and therapeutic agents is necessary. Many researchers are trying to search for candidates to prevent dry AMD in natural sources. However, the protective effects of *S. maxima* against BL-caused retinal damage have not been demonstrated. *Spirulina maxima* (*S. maxima*) is a cyanobacterium containing phycobiliprotein, vitamins, carotenes, and phenolic compounds [21]. *S. maxima* has been reported to have a considerable antioxidant effect in several studies in vitro and in vivo [22,23]. The various compounds in *S. maxima* exert several health-promoting properties, including anti-inflammatory effects [22], hypolipidemic effects [23], and anti-neurotoxicity [24], as well as antioxidant effects. *S. maxima* is considered to be an edible nutraceutical and a functional food ingredient without toxicity by Korea Food and Drug Administration [25]. Therefore, we aimed to demonstrate the protective effects of *S. maxima* and the involved mechanisms in a blue light-induced macular degeneration model in vitro and *in vivo*.

## 2. Materials and Methods

### 2.1. Preparation of S. maxima and P-Phycocyanin

*S. maxima* was obtained from the Korea Institute of Ocean Science and Technology (KIOST, Jeju Research Center, Busan, Korea). *S. maxima* was harvested as follows: *S. maxima* was inoculated into the open raceway system (ORP) of microalgae cultivation plants using spirulina medium and cultured for one month. Cultured *S. maxima* was harvested by centrifugation using a tubular separator (Thermo Fisher Scientific, Waltham, MA, USA). Then, the harvested *S. maxima* was stored at −50 °C and lyophilized (Operon, Gimpo, South Korea). P-phycocyanin was prepared as previously described [26].

### 2.2. Cell Culture

Human RPE cells (American Type Culture Collection, Manassas, VA, USA) were maintained in Dulbecco’s modified Eagle’s medium F-12 (Welgene, Daegu, Republic of Korea) supplemented with 10% fetal bovine serum at 37 °C. The cells were grown in a humidified atmosphere saturated with 5% CO_2_.

### 2.3. Cell Viability Assay

ARPE-19 cells were seeded in a 96-well plate and treated with *S. maxima* (0, 50, 100, 200, 400, and 800 μg/mL) or A2E (0, 5, 10, 20, 40, and 60 μM) or P-phycocyanin (0, 4.3, 8.5, 17, 34.1, and 68.2 μM) for 24 h. Cell viability was assessed by Cell Count Kit-8 (CCK) assay (Dojindo Labs., Japan). After adding 10 μL of the CCK solution to each well, the cells were maintained at 37 °C for 1 h. Absorbance at 450 nm was recorded by ELISA reader (Bio-Tek instrument: Power Wave XS microplate spectrophotometer, Winooski, VT, USA). To assess the cytotoxicity caused by BL in A2E-loaded ARPE-19 cells, the RPE cells were seeded in a 96-well plate, treated with A2E (0, 5, 10, 20, 40, and 60 μM), exposed to BL (430 nm, 6000 lux, 10 min) and incubated for an additional 24 h. Then, cell viability was measured by the CCK assay.

### 2.4. Pre and Post-Treatment of S. maxima with BL Exposure

ARPE-19 cells were seeded in 96-well plates at a density of 1.5 × 10^4^ cells per well and grown for 24 h. For post-treatment system, the ARPE-19 cells were treated with 20 μM A2E and incubated for 24 h. After the supernatant was suctioned, the cells were treated with *S. maxima* (50, 100, and 200 μg/mL) or lutein (30 μM) or P-phycocyanin (8.5, 17, and 34.1 μM) and then maintained for 24 h. For the pre-treatment system, the RPE cells were treated with *S. maxima* (50, 100, and 200 μg/mL) or lutein (30 μM) or P-phycocyanin (8.5, 17, and 34.1 μM) for 24 h. After the supernatant was removed, the cells were treated with 20 μM A2E for 24 h. In both systems for sample treatment, the supernatant was replaced with PBS and blue light exposure (430 nm, 6000 lux, 10 min) was performed in ARPE-19 cells. After the cells were maintained for 24 h, cell viability was assessed using CCK assay. This experimental condition was for efficacy screening *in vitro*.

### 2.5. A2E Treatment and Blue Light Exposure

The ARPE-19 cells were seeded at the density of 5 × 10^4^ cells per well in 6-well plates. The cells were treated with 20 μM A2E three times (day 1, 3, and 5) for 6 days, and treated with *S. maxima* (100 μg/mL) or lutein (30 μM) twice (day 7 and 9) for 4 days. Next, the cells were irradiated with BL (430 nm, 6000 lux, 20 min) and maintained for an additional 6 h. This experimental condition was for underlying mechanism study *in vitro*.

### 2.6. Estimation of Cellular Reactive Oxygen Species (ROS)

After the treated cells were irradiated with BL and incubated for 6 h, the RPE cells were harvested and subjected to ROS quantification by OxiSelect in vitro ROS/RNS assay kit from Cell Biolabs (Sandiego, CA, USA). According to the manufacturer’s protocol, the cells were suspended in PBS, sonicated on ice, and centrifuged at 10,000× *g* for 5 min. The supernatant was added with 2’,7’-dichlorofluorescin (DCFH) solution in a 96-well plate for 30 min at room temperature. The fluorescence intensity of DCF was assessed by a fluorescence plate reader at 480 nm excitation and 530 nm emission.

### 2.7. Animals and Experiment Design

Five-week-old male Balb/c mice were obtained from DBL (Cheongju, Republic of Korea). All the mice were housed in a room on a 12:12 h light-dark cycle at 25 ± 1 °C and were freely fed water and standard laboratory diets (Central Lab Animal, Seoul, Korea). All procedures were approved by the Institutional Animal Care and Use Committee guideline of Kyung Hee University [KHUASP(SE)-19-036]. The mice were randomly separated into six groups (*n* = 6 per group). All samples including lutein and *S. maxima* were dissolved in 0.5% carboxyl methylcellulose (CMC). Normal group: mice administered with 0.5% CMC (as the vehicle). Blue light-exposed group: mice exposed to blue light and administered with 0.5% CMC. Lutein-treated group: mice exposed to blue light and administered with lutein (100 mg/kg body weight). *S. maxima* treated groups: mice exposed to blue light and administered with *S. maxima* (50, 100, 200 mg/kg body weight).

### 2.8. Sample Administration and BL Exposure

The sample administration and BL exposure was performed in accordance with the schedules established in our previous study [17]. Before blue light exposure was performed, lutein or *S. maxima* was intragastrically administered to the mice every 24 h for 5 days. After dark adaptation for 24 h, sample administration and blue light exposure were conducted every 24 h for 14 days. One hour after the gastric administration the mice, except for normal group, were exposed to blue light (430 nm, 10,000 lux) for 1 h. After the 14th blue light exposure to the mice, all the mice were housed in a dark room for 24 h and were euthanized by a CO_2_ chamber.

### 2.9. Histological Analysis

After euthanasia, eyeballs were immediately enucleated and fixed in Davidson’s solution (10% neutral buffered formaldehyde: 95% ethanol: glacial acetic acid: distilled water = 1:3:1:3) for 7 days. The entire eyes were embedded in paraffin and were cut along the vertical meridian of eyeballs. The paraffin-embedded sections were stained with hematoxylin and eosin (H&E) and photographed using an optical microscope (Olympus Optical, Tokyo, Japan). The thickness of the outer nuclear layer (ONL), inner nuclear layer (INL), photoreceptor layer (PL), and the whole retina were measured between 600 and 900 μm from the optic disc at 60 μm intervals using image J software (National Institutes of Health, Bethesda, MD, USA). The average thickness was calculated from 6 locations for each eye.

### 2.10. Quantitative Real Time PCR (qRT-PCR)

Total RNA from the treated cells and retina tissues was extracted using Easy-RED reagent (iNtRON biotechnology, Seongnam, Republic of Korea). The extracted RNA samples were used to synthesize cDNA using a cDNA synthesis kit (TaKaRa, Tokyo, Japan). The expressions of genes were analyzed using an ABI StepOnePlus™ Real-Time PCR System (Applied Biosystems, Foster City, CA, USA). The qRT-PCR was operated according to the manufacturer’s protocols and SYBR Premix Ex Tag (TaKaRa, Tokyo, Japan) was used as the dye. The primer sequences for qRT-PCR were listed in Table 1. The gene expressions were calculated relative to *GAPDH*.

### 2.11. Western Immunoblotting

ARPE-19 cells were collected and lysed in lysis buffer containing cOmplete^™^ Protease Inhibitor Cocktail tablets (Roche Diagnostics, Indianapolis, IN, USA). The lysates were sonicated for 20 min and centrifuged at 10,000 g for 20 min, and the supernatants were obtained. Nuclear and cytosol fractions of the protein were separated by a Nuclear Extraction Kit (Abcam, Cambridge, MA, USA) according to the manufacturer’s instructions. The concentrations of protein were measured with Pierce™ BCA Protein Assay Kit from Thermo Fisher Scientific (Rockford, IL, USA). According to the manufacturer’s protocol, we loaded approximately 1000–1500 μg/mL protein from the cytosol and 500–1000 μg/mL protein from the nucleus in both ARPE-19 cells and mice retina. The equal amount of protein was applied to sodium dodecyl sulfate polyacrylamide gel and the proteins were transferred to a polyvinylidene fluoride membrane. The membranes were blocked with 5% skim milk and incubated with a primary antibody at 4 °C overnight, followed by incubation with a horseradish peroxidase (HRP)-conjugated secondary antibody for 2 h. The membranes were visualized by a LAS3000 Luminescent image analyzer (Fuji Film, Tokyo, Japan). The protein band intensities were measured using the Image J software and normalized to β-actin. Lamin B1 was used to normalize for NF-κB p65 in the nucleus. Antibodies for NF-κB p65, IκB-α, caspase 3, and β-actin were obtained from Santa Cruz Biotechnology (Delaware, CA, USA). Antibodies for PARP, and Lamin B1 were obtained from Cell Signaling Technology (Danvers, MA, USA).

### 2.12. Statistical Analysis

Shapiro–Wilk tests were performed to analyze the normality of the data. Normally distributed data were cell viability tests, sample pre- and post-treatment tests, and histological analysis of the retinal layer thicknesses. One-way ANOVA followed by Tukey’s post-hoc tests were used to assess statistical significance for normally distributed data. Mann–Whitney tests were used to analyze the non-normally distributed data. Significant differences were assessed using SPSS version 25 statistical software (Chicago, IL, USA).

## 3. Results

### 3.1. S. maxima Inhibited Cell Death Caused by A2E Treatment and BL Exposure

**Figure 1:**
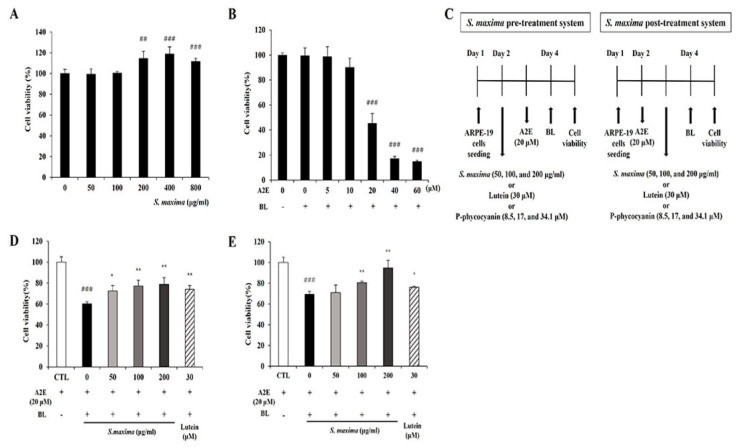
*S. maxima* inhibited cell death caused by A2E treatment and BL exposure. (**A**) Cytotoxicity of *S. maxima* in RPE cells was assessed by CCK assay. (**B**) RPE cells were treated with A2E at the concentration of 0, 5, 10, 20, 40, 60 μM for 24 h. After BL (430 nm, 6000 lux, 10 min) exposure in the cells and then incubation for 24 h, cytotoxicity was assessed by the CCK assay. (**C**) Treatment schedule for pre-and post-treatment systems. (**D**) Sample pre-treatment system: the ARPE-19 cells were treated and maintained with *S. maxima* (0–200 μg/mL) or lutein (30 μM) for 24 h. Then, the cells were treated with A2E (20 μM) for 24 h. After BL (430 nm, 6000 lux, 10 min) exposure in the cells and then incubation for 24 h, cell viability was measured by the CCK assay. (**E**) Sample post-treatment system: the ARPE-19 cells were treated with A2E (20 μM) for 24 h, then the cells were treated and maintained with *S. maxima* (0–200 μg/mL) or lutein (30 μM) for 24 h. After BL (430 nm, 6000 lux, 10 min) exposure in the cells and then incubation for 24 h, cell viability was measured by the CCK assay. The results were shown as the mean ± SD (*n* = 3) of the three independent experiments. ^##^ *p* < 0.01, ^###^ *p* < 0.001 vs. CTL. * *p* < 0.05, ** *p* < 0.01 vs. BL.

The RPE cells were treated with various concentrations of *S. maxima* (0, 50, 100, 200, 400, and 800 μg/mL), and *S. maxima* did not show cytotoxicity. Moreover, the cell viability was increased at the concentration of 200, 400, and 800 μg/mL (Figure 1A). Cell viability was maintained when the RPE cells were treated with 10 μM A2E. However, treatment of 20 μM A2E caused a reduction in cell viability of 54.4% when the cells were irradiated with BL (Figure 1B). Pre- and post-treatment of the cells with *S. maxima* significantly inhibited BL-caused cell death in a concentration-dependent manner (Figure 1C,D). The treatment of *S. maxima* at concentrations of 100 and 200 μg/mL exerted more preventive effects against RPE cell death by BL than lutein (30 μM) did. After efficacy tests of *S. maxima* at various concentrations, we determined 100 μg/mL of *S. maxima* for the following mechanism study.

### 3.2. S. maxima Regulated the Inflammatory Response Caused by BL in A2E-Laden ARPE-19 Cells

**Figure 2:**
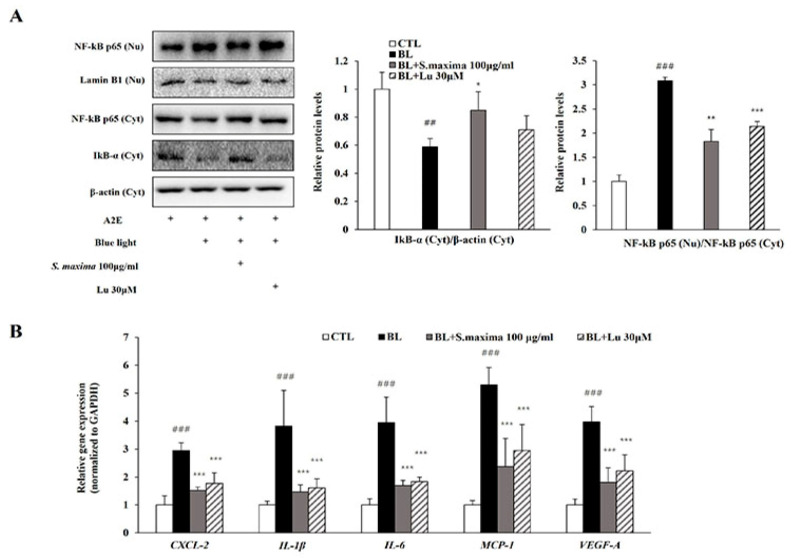
*S. maxima* regulated the inflammatory response caused by BL in A2E-laden ARPE-19 cells. (**A**) Effects of *S. maxima* on protein levels in A2E-loaded RPE cells were estimated by western immunoblotting. NF-κB p65 (65 kDa) was estimated in nucleus and cytosol fractions. IκBα (37 kDa) was measured in cytosol fractions only. Protein levels of NF-κB p65 (65 kDa) and IκBα (37 kDa) were quantified by band density. The results were presented as the mean ± SD (*n* = 3) of three independent experiments. (**B**) Gene expressions related to inflammation were measured by quantitative real time PCR in A2E-laden ARPE-19 cells and normalized to GAPDH. The results were shown as the mean ± SD of three independent experiments (*n* = 4). ^##^ *p* < 0.01, ^###^ *p* < 0.001 vs. CTL, * *p* < 0.05, ** *p* < 0.01, *** *p* < 0.001 vs. BL.

It is well known that BL exposure induces A2E photo-oxidation and cell death [2,7]. Our previous studies demonstrated that oxidative stress caused by blue light activated nuclear factor kappa B (NF-κB) pathways [17]. NF-κB is a kind of transcription factor contributed to the inflammation. To examine whether *S. maxima* regulates NF-κB activation by BL exposure, we measured gene and protein expressions. In the cytosol, IκB-α was associated with NF-κB and the blocks functions of NF-κB as a transcription factor. When an inflammatory response occurred, IκB-α was degraded and reduced. Then, NF-κB is activated and translocated to the nucleus. When A2E-loaded RPE cells were exposed to BL, degradation of IκB-α was increased, and protein level was decreased (Figure 2A). NF-κB translocation to the nucleus was increased by BL exposure, causing an increased NF-κB protein level in the nucleus. However, treatment of *S. maxima* inhibited the translocation of NF-κB and reduced the nuclear protein level of NF-κB compared to that in BL-exposed ARPE-19 cells.

Activated NF-κB acts as a transcription factor in the nucleus, causing the transcription of several cytokines like *CXCL-2*, *IL-1β*, and *IL-6*. BL exposure upregulated the gene expressions of cytokines, including *CXCL-2*, *IL-1β*, *IL-6*, and *MCP-1* involved in inflammation (Figure 2B). In addition, the expression of *VEGF-A*, contributing to the progression to wet AMD, was increased by BL exposure, suggesting that angiogenesis was induced. However, we observed that the gene expressions of the cytokines were significantly attenuated by *S. maxima* treatment. These results indicate that *S. maxima* decreases inflammation caused by BL through regulating inflammatory-related gene expressions. *S. maxima* also prevented the progression from dry AMD to wet AMD by suppressing the expressions of *VEGF-A*.

### 3.3. S. maxima Regulated the Apoptosis Caused by BL in A2E-Laden ARPE-19 Cells

**Figure 3:**
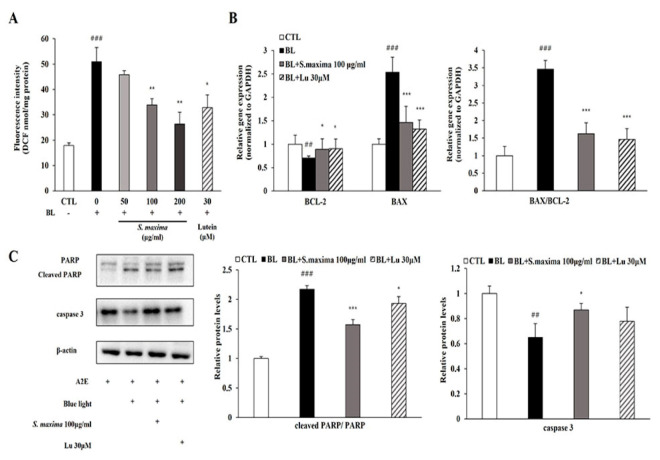
*S. maxima* regulated the apoptosis caused by BL in A2E-laden ARPE-19 cells. (**A**) Estimation of ROS/RNS levels in A2E-laden ARPE-19 cells. The results were shown as the mean ± SD (*n* = 3) of three independent experiments. ^###^ *p* < 0.001 vs. CTL, * *p* < 0.05, ** *p* < 0.01 vs. BL. (**B**) Gene expressions related to apoptosis were measured by quantitative real time PCR in A2E-loaded RPE cells and normalized to GAPDH. The results were shown as the mean ± SD of three independent experiments, *n* = 4 per group. (**C**) Protein levels of PARP (116 kDa), cleaved PARP (89 kDa), caspase 3 (32 kDa) were estimated by Western immunoblotting. Protein levels were quantified by band density. The results were shown as the mean ± SD (*n* = 3) of three independent experiments. ^##^ *p* < 0.01, ^###^ *p* < 0.001 vs. CTL, * *p* < 0.05, *** *p* < 0.001 vs. BL.

We hypothesized that *S. maxima* might prevent apoptosis by reducing ROS generation after BL exposure. The total ROS/RNS level was significantly increased after BL exposure and restored by the treatment of *S. maxima* in a concentration-dependent manner (Figure 3A).

To investigate the regulatory effects of *S. maxima* on apoptosis pathways, we measured mRNA and the protein levels of the related genes. *BCL-2,* an anti-apoptotic protein, showed decreased mRNA expression, whereas *BAX,* a pro-apoptotic protein, showed increased mRNA expression after BL exposure, resulting in a raised *BAX/BCL-2* ratio (Figure 3B). Then, we investigated the regulatory effects on caspase 3 and PARP activation by *S. maxima*. The protein level of caspase 3 was decreased when caspase 3 cleavage occurred. Activated caspase 3 induced PARP cleavage, increasing the ratio of cleaved PARP/full-length PARP by BL exposure in Figure 3C. Increased *BAX/BCL-2* ratio was restored by *S. maxima* treatment, indicating a decreased mitochondrial apoptosis. Caspase 3 activation and PARP cleavage were decreased by *S. maxima* treatment compared with the BL-exposed group.

### 3.4. S. maxima Protected Photoreceptor Degeneration Caused by BL in Retina

**Figure 4:**
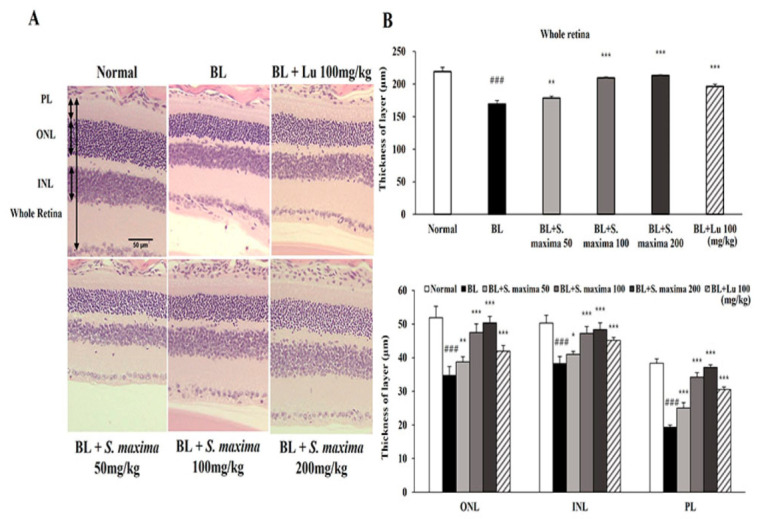
*S. maxima* protected photoreceptor degeneration caused by BL in retina. (**A**) Representative retinal images that were H&E staining. Scale bar = 50 μm. (**B**) Whole retina, ONL; outer nuclear layer, INL; inner nuclear layer, PL; photoreceptor layer thicknesses were measured at six different locations and averaged. The results were shown as mean ± SD (*n* = 6). ^###^ *p* < 0.001 vs. Normal, * *p* < 0.05, ** *p* < 0.01, *** *p* < 0.001 vs. BL-exposed group.

We found that *S. maxima* exerted protective effects on BL-caused damage in RPE cells. To investigate whether *S. maxima* had inhibitory effects against photoreceptor degeneration, we performed the histological analysis in our established animal model [17]. Retina images are presented between 600 and 900 μm from the optic nerve in Figure 4A. The thicknesses of all layers (whole retina, ONL, INL, and PL) were remarkably reduced in the BL-exposed group compared with the normal group (Figure 4B). However, the reductions in thicknesses were suppressed by the administration of *S. maxima.* These observations suggest that *S. maxima* prevents BL-caused retinal degeneration in the retina.

### 3.5. S. maxima Regulated Inflammation and Apoptosis Caused by BL in Retina

**Figure 5:**
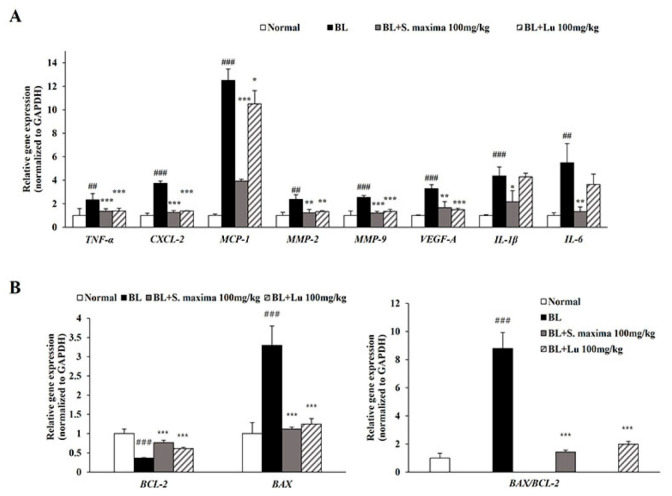
*S. maxima* regulated the inflammation and apoptosis caused by BL in retina. (**A**) The total RNA was isolated from mice retinas. Gene expressions related to inflammation (*TNF-α, CXCL-2, MCP-1, MMP-2, MMP-9, VEGF-A, IL-1β,* and *IL-6*) were measured in retinas using quantitative real time PCR. (**B**) Gene expressions related to apoptosis (*BCL-2*, and *BAX*) were analyzed in retinas using quantitative real time PCR. The results are shown as mean ± SD (*n* = 4). ^##^ *p* < 0.01, ^###^ *p* < 0.001 vs. Normal, * *p* < 0.05, ** *p* < 0.01, *** *p* < 0.001 vs. BL exposed group.

In this study, BL exposure upregulated expressions of inflammation-related genes (*CXCL-2*, *IL-1β*, *IL-6*, and *MCP-1*) and *VEGF-A* in the ARPE-19 cells. To correlate with in vitro study, we investigated expressions of inflammation-related genes (*TNF-α*, *CXCL-2*, *IL-1β*, *IL-6*, and *MCP-1*) and angiogenesis-related genes (*MMP-2*, *MMP-9*, and *VEGF-A*) in mice retina (Figure 5A). BL illumination increased the expressions of inflammatory-related genes in mice retina. However, the expressions were attenuated by the administration of *S. maxima*. We demonstrated that BL exposure induced apoptosis by increasing *BAX* level and decreasing *BCL-2* level, whereas *S. maxima* regulated apoptosis caused by BL in A2E-loaded RPE cells. To correlate with the in vitro study, we investigated the expressions of apoptosis-related genes (*BAX, BCL-2*) in mice retina. BL exposure decreased the gene expression of *BCL-2* and raised *BAX* expression, resulting in elevating the *BAX/BCL-2* ratio (Figure 5B). However, the *BAX/BCL-2* ratio was reduced by *S. maxima* administration.

### 3.6. P-Phycocyanin Was a Major Active Component of S. maxima on Retinal Degeneration

**Figure 6:**
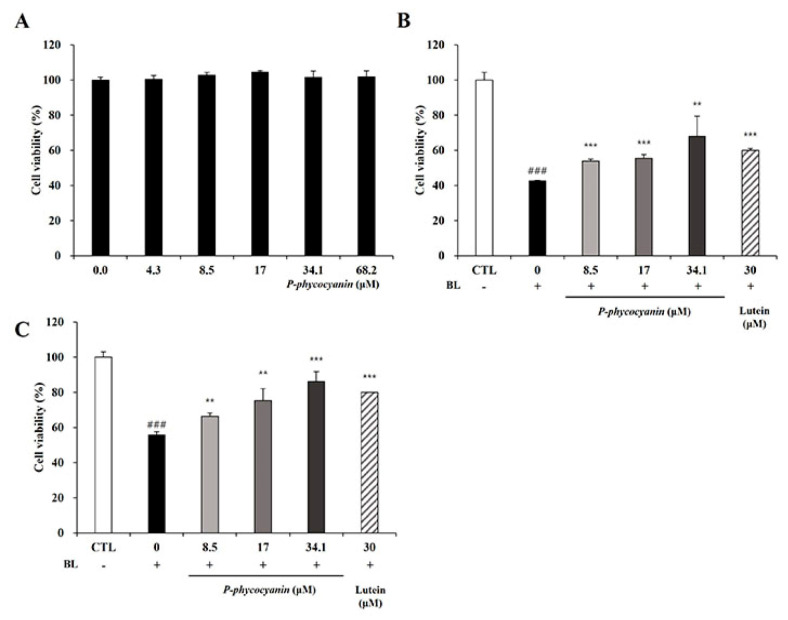
P-phycocyanin was a major active component of *S. maxima* on retinal degeneration. (**A**) Cytotoxicity of P-phycocyanin in RPE cells was assessed using the CCK assay. The results are shown as the mean ± SD (*n* = 4) of the three independent experiments. (**B**) Pre-treatment system: ARPE-19 cells were incubated with P-phycocyanin (0–34.1 μM) or lutein (30 μM) for 24 h. Then, the RPE cells were treated with A2E at 20 μM for 24 h. After the cells were irradiated with blue light (6000 lux) for 10 min and then incubated for 24 h, cell viability was assessed by the CCK assay. (**C**) Post-treatment system: ARPE-19 cells were treated with A2E at 20 μM for 24 h. Then, the RPE cells were incubated with P-phycocyanin (0–34.1 μM) or lutein (30 μM) for 24 h. After the cells were irradiated with BL (6000 lux) for 10 min and then incubated for 24 h, cytotoxicity was measured using the CCK assay. The results were shown as mean ± SD (*n* = 3) of three independent experiments. ^###^ *p* < 0.001 vs. the untreated control. ** *p* < 0.01, *** *p* < 0.001 vs. the blue light only treated group.

The pre-and post-treatment systems were conducted to elucidate whether P-phycocyanin, the most abundant ingredient in *S. maxima*, was one of the active components of *S. maxima* on retinal degeneration. We evaluated the cytotoxicity of P-phycocyanin in RPE cells to examine its efficacy within non-toxic concentrations. P-phycocyanin did not show cytotoxicity up to 68.2 μM (=40 μg/mL) in ARPE-19 cells (Figure 6A). P-phycocyanin at the concentration of 8.5, 17, 34.1 μM (=5, 10, 20 μg/mL) inhibited BL-induced cell death concentration dependently in the pre- and post-treatment systems (Figure 6B,C). In the pre-treatment system, the protective effects of P-phycocyanin at 34.1 μM were comparable with *S. maxima* of 200 μg/mL (Figure 1C and Figure 6B). Post-treatment of P-phycocyanin at 8.5, 17, and 34.1 μM exerted similar protective effects with *S. maxima* at concentrations of 50, 100, and 200 μg/mL, respectively (Figure 1D and Figure 6C). These results indicate that most of the efficacy of *S. maxima* is due to P-phycocyanin. Thus, we demonstrated that P-phycocyanin was the main active compound of *S. maxima* in RPE cells.

## 4. Discussion

A2E accumulates in lysosomes of RPE cells with aging, contributing to the development of AMD. A2E is non-toxic to RPE cells at low concentrations, but it is highly toxic when A2E-loaded RPE cells are irradiated with BL at 430 nm [27]. Exposure to BL (430 nm) is considered one of the key factors leading to RPE damage, followed by cell death [28]. Our results showed that cytotoxicity was caused when 20 μM A2E-loaded RPE cells were exposed to BL (Figure 1B). Therefore, we investigated the efficacy and underlying mechanisms of *S. maxima* on retinal damage using 20 μM A2E and BL (430 nm) irradiation.

We confirmed the efficacy of *S. maxima* on BL-caused retinal damage *in vitro*. Pre- and post-treatment of *S. maxima* inhibited RPE cell death concentration dependently (Figure 1C,D). The previous study demonstrated that high levels of oxidative stress were caused by ROS generation when exposed to BL in A2E-laden RPE cells. Oxidative stress is related to the pathogenesis and progression of AMD [29]. Therefore, we estimated ROS levels in RPE cells. BL exposure increased the ROS levels in the cells, whereas *S. maxima* significantly suppressed the levels (Figure 3A). *S. maxima* protected RPE cell death from oxidative stress by reducing ROS generation.

To investigate the action mechanisms of *S. maxima*, this study focused on NF-κB signaling, inflammatory response, and the apoptosis pathway. The inflammatory response is one of the major causes contributing to the AMD pathogenesis [30]. The NF-κB activation leads to increases in transcription of pro-inflammatory cytokines such as *IL-1β* and *IL-6* [17,31]. CXCL-2, as a chemokine and a target gene of NF-κB, modulated the immune response by chemoattracting neutrophils and was upregulated when exposed to light in ARPE-19 cells [20,32]. MCP-1 acts as a chemoattractant for neutrophils and macrophages. MCP-1 is mediated by the activation of NF-κB, contributing to angiogenesis [33]. VEGF-A, a critical regulator of angiogenesis, is related to wet AMD development [34]. BL exposure increased the NF-κB translocation into the nucleus in RPE cells, leading to upregulation of the expressions of inflammation-related genes (*IL-1β*, *IL-6*, *CXCL-2*, and *MCP-1*) and *VEGF-A* (Figure 2A,B). Treatment of *S. maxima* attenuated the expressions of the genes by inhibiting IκB-α degradation and NF-κB translocation at the concentration of 100 μg/mL in RPE cells. Moreover, *S. maxima* treatment significantly decreased VEGF-A expression as a pro-angiogenic marker, suggesting that *S. maxima* suppressed the advancement of dry AMD to wet AMD. This suggests that *S. maxima* significantly suppresses the inflammatory response via modulating the NF-κB pathway.

Previous studies have reported that BL exposure causes A2E oxidation and generates reactive oxygen species (ROS), including hydrogen peroxide and superoxide anion [13]. Generated ROS causes oxidative stress to cells, leading to apoptosis [35]. Oxidative stress and inflammation contribute to apoptosis, related to AMD pathogenesis [36]. *BAX* and *BCL-2* serve as critical regulators of mitochondrial apoptosis. BAX, a pro-apoptotic protein, resides in the mitochondrial outer membrane and induces the release of cytochrome c from mitochondria, followed by inducing caspase activation in the cytosol. BCL-2 regulates cell death by suppressing apoptosis and other proteins such as BAX that cause cell death. Moreover, the overexpression of BCL-2 inhibits the release of cytochrome c and the activation of caspases [37]. The release of cytochrome c leads to activation of caspase 3 and the cleavage of PARP, which is considered a marker of apoptosis [38]. BL exposure increased the ratio of *BAX/BCL-2*, followed by decreasing the protein levels of caspase 3 and increasing cleaved PARP levels in RPE cells, which was restored by *S. maxima* treatment (Figure 3B,C). These results imply that *S. maxima* inhibits RPE cell death by regulating the apoptotic pathway.

We estimated the efficacy of *S. maxima* in our animal model of retinal degeneration by analyzing the thickness of each retinal layer (ONL, INL, and PL). ONL consists of the photoreceptor cells. The thickness of ONL is mainly measured to evaluate the photoreceptor degeneration [39]. INL, which is contained in the horizontal, bipolar, and amacrine cells, contributes to light signal transduction [40]. PL, outer and inner segments of photoreceptors, are comprised of photoreceptors, such as rods and cones [41]. The administration of *S. maxima* suppressed retinal degeneration dose-dependently by restoring the reduction of the thickness of all three layers (Figure 4A,B). Unlike the in vitro study that focused on RPE cells, our in vivo study investigated whole retinal degeneration, which was verified by analyzing the thickness of each retinal layer. However, further studies are needed to examine the RPE alterations and the degeneration in the retina. Based on these findings, we demonstrated the relevant mechanisms of *S. maxima* using the mouse retina. Microglia activation induced by light exposure is related to the inflammatory response and is involved in AMD pathogenesis [42]. Reactive microglia accumulate in the ONL and subretinal space and release pro-inflammatory cytokines such as TNF-α, IL-1β, and IL-6 [43,44]. Therefore, we confirmed the expression change of the inflammatory-related genes. TNF-α, a pro-inflammatory cytokine, is produced by macrophages [30]. *S. maxima* downregulated the expressions of inflammatory-related genes (*TNF-α*, *CXCL-2*, *MCP-1*, *IL-1β*, and *IL-6*) induced by BL exposure. The findings suggest that the administration of *S. maxima* prevents BL-induced retinal degeneration via regulating the expressions of inflammation-related genes. MMP-2 and MMP-9 are extracellular proteinases related to neovascularization and increased vascular permeability in the retina [45]. The previous study reported that gene expressions of *MMP-2* and *MMP-9* were upregulated in the retina by light exposure [20]. We found that BL exposure induced the gene expressions of *MMP-2* and *MMP-9*. However, the administration of *S. maxima* significantly recovered expressions of these genes to the normal levels (Figure 5A). The gene expression of *VEGF-A* was decreased by *S. maxima*, indicating that *S. maxima* prevented the advancement of dry AMD to wet AMD. Substances that could inhibit the advancement to wet AMD have not been reported. The ratio of *BAX/BCL-2* was significantly decreased in the *S. maxima*-treated group compared with the BL-exposed group, suggesting that *S. maxima* suppressed apoptosis in the retina (Figure 5B). These results were correlated with in vitro study.

*S. maxima* contains phycocyanin, which is a pigment–protein complex [46]. P-phycocyanin is one of the phycocyanin species in *S. maxima*. The P- means that the prefix, derived from “pan”, was chosen to indicate the wide distribution of biliprotein [47]. Our previous study demonstrated that the main components of *S. maxima* were identified as P-phycocyanin α-subunit and β-subunit [26]. We investigated whether P-phycocyanin was the major active ingredient in *S. maxima* on retinal degeneration. Phycocyanin, one of the pigment constituents in Spirulina, has anti-inflammatory and antioxidant activities [48]. Several studies have reported that C-phycocyanin decreased the generation of alkoxy and hydroxyl radical in vitro, and glucose oxidase-induced inflammation in mouse paws [49]. *S. maxima* did not show cytotoxicity up to 800 μg/mL concentration. Therefore, in Figure 1C,D, we selected 50, 100, and 200 μg/mL concentrations of *S. maxima* for the efficacy study of *S. maxima* in A2E-laden ARPE-19 cells. P-phycocyanin is contained about 10% in *S. maxima*. So, we selected 8.5, 17, and 34.1 μM (=5, 10, and 20 μg/mL) concentrations of P-phycocyanin for the efficacy study of P-phycocyanin in A2E-laden ARPE-19 cells. P-phycocyanin at 34.1 μM (=20 μg/mL), exhibited similar protective effects with *S. maxima* at 200 μg/mL concentration in the pre-and post-treatment systems (Figure 6B,C). Therefore, this study suggests that P-phycocyanin is the main active component of *S. maxima*.

## 5. Conclusions

We demonstrated that *S. maxima* exerts protective effects on BL-induced cell death via regulating ROS production. *S. maxima* had anti-inflammatory and anti-apoptotic effects through modulating the NF-κB pathway in RPE cells. The administration of *S. maxima* inhibited BL-induced retinal degeneration by restoring the thicknesses of the retinal layers. *S. maxima* downregulated the expressions of genes related to inflammation and apoptosis in the mouse retina. *S. maxima* suppressed the expressions of angiogenesis-related genes (*MMP-2*, *MMP-9*, and *VEGF-A*) in the mouse retina. P-phycocyanin, the most abundant compound of *S. maxima*, was a main active ingredient of *S. maxima* on retinal degeneration. Therefore, *S. maxima* could be a potential therapeutic agent to prevent the patho-physiological processes leading to dry AMD, and the progression to wet AMD.

## Figures and Tables

**Table 1:** Primer sequences used for qRT-PCR (*in vitro/in vivo*).

Gene	Forward (5’-3’)	Reverse (5’-3’)
*Bcl-2*	ATGTGTGTGGAGAGCGTCAA	ACAGTTCCACAAAGGCATCC
*Bax*	GGGGACGAACTGGACAGTAA	CAGTTGAAGTTGCCGTCAGA
*IL-1* *β*	GGACAAGCTGAGGAAGATGC	TCGTTATCCCATGTGTCGAA
*IL-6*	CACAGACAGCCACTCACCTC	TTTTCTGCCAGTGCCTCTTT
*CXCL-2*	GGGCAGAAGCTTGTCTCAA	AGCTTCCTCCTTCCTTCTGG
*MCP-1*	ATGAAAGTCTCTGCCGCCCTCA	GAGATCTGTGCTGACCCCAA
*VEGF-A*	TTGCCTTGCTGCTCTACCTC	AAATGCTTTCTCCGCTCTGA
*GAPDH*	CGAGATCCCTCCAAAATCAA	TTCACACCCATGACGAACAT
*Bcl-2*	CTCGTCGCTACCGTCGTGACTTCG	CAGATGCCGGTTCAGGTACTCAGTC
*Bax*	AAGCTGAGCGAGTGTCTCCGGCG	GCCACAAAGATGGTCACTGTCTGCC
*IL-1* *β*	TCGCAGCAGCACATCAACAAG	TCCACGGGAAAGACACAGGTAG
*IL-6*	TGTGCAATGGCAATTCTGAT	GGTACTCCAGAAGACCAGAGGA
*CXCL-2*	CGCTGTCAATGCCTGAAGAC	ACACTCAAGCTCTGGATGTTCTTG
*TNF-α*	CACAAGATGCTGGGACAGTGA	TCCTTGATGGTGGTGCATGA
*MCP-1*	TTAAGGCATCACAGTCCGAG	TGAATGTGAAGTTGACCCGT
*MMP-2*	TGGCAAGGTGTGGTGTGCGAC	TCGGGGCCATCAGAGCTCCAG
*MMP-9*	GGTGTGCCCTGGAACTCACACG	AGGGCACTGCAGGAGGTCGT
*VEGF-A*	CCTGGTGGACATCTTCCAGGAGTACC	GAAGCTCATCTCTCCTATGTGCTGGC
*GAPDH*	CGGCCGCATCTTCTTGTG	CCGACCTTCACCATTTTGTCTAC

CXCL-2; chemokine (C-X-C motif) ligand 2, TNF-α; tumor necrosis factor alpha, MCP-1; monocyte chemoattractant protein 1, MMP-2; matrix metallopeptidase 2, MMP-9; matrix metallopeptidase 9, VEGF-A; vascular endothelial growth factor-A, Bcl-2; B-cell lymphoma 2, Bax; Bcl-2-associated X protein, GAPDH; glyceraldehyde 3-phosphate dehydrogenase.

## Data Availability

The data presented in this study are available on request from the corresponding author.
